# MicroRNAs in Acute Myeloid Leukemia and Other Blood Disorders

**DOI:** 10.1155/2012/603830

**Published:** 2012-06-17

**Authors:** Yao Yuan, Siddha Kasar, Chingiz Underbayev, Sindhuri Prakash, Elizabeth Raveche

**Affiliations:** ^1^New Jersey Medical School, Newark, NJ 08731, USA; ^2^Pathology and Laboratory Medicine, New Jersey Medical School, MSB C512, Newark, NJ 07103, USA

## Abstract

Common blood disorders include hematopoietic cell malignancies or leukemias and plasma cell dyscrasia, all of which have associated microRNA abnormalities. In this paper, we discuss several leukemias including acute myeloid leukemia (AML) and chronic lymphocytic leukemia (CLL) and identify altered microRNAs and their targets. Immune disorders with altered blood levels of antibodies include autoimmune disorders, such as systemic lupus erythematosus (SLE) with associated anti-self-autoantibodies and immunoglobulin A nephropathy (IgAN) also have related microRNA abnormalities. The alterations in microRNAs may serve as therapeutic targets in these blood disorders.

## 1. Introduction 

MicroRNAs are small (20–22 nt), evolutionarily conserved, noncoding single-stranded RNAs discovered in the 1990s [1, 2], functioning to target 3′ untranslated region (UTR) of mRNAs in antisense sequence specific way and regulate genes posttranscriptionally for degradation or translation suppression. MicroRNAs target 1–3% of all eukaryotic genes yet regulating ~30% of protein-coding genes [3]. The miRNAs are first transcribed by RNA polymerase II in the nucleus as large primary transcript (pri-miRNA) [4], either from independent genes or from clustered genes encoding several miRNAs [5] and further processed into ~70 nt pre-miRNA with hairpin structure by Drosha, a RNase III type endonuclease (RN3) in the nucleus. Alternatively, in the nucleus, a small class of “mintron” without the stem-loop and the flanking single-strand structure as in pri-miRNA required for Drosha processing, could be generated by passing Drosha-dependent pathway [6]. In the cytoplasm, ~20 bp miRNA/miRNA* duplex are generated by Dicer, another RN3 endonuclease. One of the miRNA duplex strands is further incorporated into protein-RNA complex called RNA-induced silencing complex (RISC), although in some cases, both arms of the pre-miRNA hairpin could generate mature miRNAs [7–9]. miRNAs interact with target mRNA by sequence complementarity, and in perfect base pairing usually triggers endonucleolytic mRNA cleavage [10]; however, in most situations, such base pairing is imperfect, resulting in translational suppression. The key component of this RISC machinery is Ago protein family (Ago 1–4), but only Ago 2 is known to have the catalytic enzyme function [11, 12]. Besides Ago proteins, GW182 protein is also recruited to the RISC complex and together localize in cytoplasmic foci called processing bodies (P bodies or GW bodies), where mRNA is sequestered from being translated [13–16]. There are different experimental and bioinformatics approaches to predict miRNA targets. At a minimum, the precise matching to 3′UTR of mRNA in multiple copies should be within the first 2–8 bases from the 5′ end of the mature miRNA, called the “seed region” [17–20]. To date, over 2000 human miRNAs have been annotated in the Sanger miRBASE (Release 18, http://www.mirbase.org/cgi-bin/browse.pl?org=hsa). The miRNA network is highly redundant, since a single miRNA may have multiple target mRNAs, and in turn, a single mRNA could be targeted by many miRNAs [21]. Various miRNAs have been shown to be involved in a myriad of cellular processes including differentiation, metabolism, apoptosis, and development [22]. Physiologically, and pathologically, miRNAs have been reported to play roles in cancers, inflammatory responses, diabetes, and autoimmunity [23, 24].

## 2. MicroRNAs in Hematopoietic Stem Cells

Multiple evidence suggest that microRNAs play a significant role in the posttranscriptional genetic regulation in stem and progenitor cells. They are involved in a number of hematological malignancies such as acute lymphoblastic leukemia, acute myeloid leukemia, chronic lymphocytic leukemia, chronic myelogenous leukemia, diffuse large-B-cell lymphoma, and others [25]. Therefore, miRNA profiling is critical in order to distinguish stem cells of the different origins, developmental stages, and genetic conditions [26]. Furthermore, it can help classify cancer cell samples and develop appropriate therapeutic strategies [27]. Recent studies have demonstrated a causative role for miRNAs in malignant diseases development in the hematopoietic system. For instance, overexpression of miR-155 or miR-29a in the mouse hematopoietic system leads to a myeloproliferative disorder [28] or leukemia [29], respectively. On the other hand, tumor suppressor miRNAs such as miR-15a/16-1 are found to be deleted in a subset of lymphomas [30] and have been shown to cause chronic lymphocytic leukemia in mice [31, 32]. MicroRNA-125b has been demonstrated to cause pathological myeloid cells expansion in a dose-dependant manner [33], and miR-155 is known to induce polyclonal expansion followed by B-cell malignancy development [34]. In another study on human umbilical cord blood, two particular miRNAs-hsa-miR-520h and hsa-miR-526b*- levels appeared to be elevated. Interestingly, ABCG2, an important factor of stem cells maintenance, is a known target of hsa-miR-520h [35].

## 3. MicroRNAs in the Immune System

Proper regulation of immune response is critical in preventing immunopathology and autoimmune disorders. Studies have implicated important functions of miRNA on hematopoietic development as well as innate and adaptive immune responses. Toll-like receptor (TLR) signaling leads to transcriptional activation of a large class of proinflammatory cytokines as well as multiple miRNAs. For example, miR-146a and miR-155 have been shown to be upregulated upon exposure to LPS in the monocytic leukemia cell line THP-1. More importantly, two key components of TLR4 signaling pathway, TRAF6 and IRAK1 have been verified to be targets for miR-146a [36]. This study for the first time profiled the miRNAs alterations in TLR signaling and proposed the miRNAs as negative regulators of TLR activation. MiR-155 is another well-studied microRNA reported to be activated by several TLR pathways [36, 37], and its negative regulatory role during TLR-mediated activation has also been addressed [38, 39]. More interestingly, IL-10 is shown to inhibit TLR-induced miR-155 [40]. To understand global miRNAs' importance in B and T development, studies were performed in which knocking out Dicer at different stages of B and T development resulted in blockage of further differentiation [41–43]. In addition, miR-155 has been found to be one of the most important miRNAs in both B and T cells as well as antigen presentation by dendritic cells (DCs) and is required for normal germinal center (GC) response [44, 45], B-cell class switching [46], Th1/Th2 polarization, and Treg development both in the thymus and peripheral [47].

## 4. MicroRNA in Autoimmune Diseases

Considering the importance of miRNAs in the immune system raises the question whether or not there is direct link between miRNAs abnormalities and immune disorders or autoimmune diseases. Interestingly, the discovery in 2002 of GW bodies (GWBs), where miRNA-mRNA reside for degradation was from serum from an autoimmune patient with motor and sensory neuropathy [48]. Subsequently, anti-GWB autoantibodies in the serum have been identified from patients with various autoimmune disorders [49], indicating an involvement of general miRNA pathway and autoantibody production. Dysregulated miRNA expression has been associated with autoimmunity, for example, miR-146a was underexpressed in PBMC from SLE patients when compared with healthy control. The study further showed that miR-146a is a negative regulator of type I interferon (IFN) pathway by targeting interferon regulatory factor (IRF) 5 and signal transducers and activators of transcription (STAT) 1, thus the decrease in miR-146a may contribute to the increased type I IFN signaling pathway observed in SLE [50]. A recent study in murine models (MRL-lpr, C57BL/6-lpr, and NZB/NZW F1) of SLE using a combination of microarray and real-time RT-PCR approaches, Dai et al. identified that miR-182-96-183 cluster, miR-31, and miR-155 are among those consistently upregulated miRNAs across different genetic background strains of mice [51]. In addition to important contribution of miR-155 to physiological immune response, its activity in autoimmune circumstances was also investigated. A murine experimental autoimmune encephalomyelitis (EAE) model with *mir-155* ^−/−^ was shown to be resistant to EAE pathology. Thus, unregulated miR-155 may be a link between inflammation and cancer via inducing a high proliferation rate resulting in increased mutations [52]. The miR-17-92 cluster locating in human chromosome 13q31 is known as an onco-miR, and this genomic region is often amplified in lymphomas and other cancer, and the mature miR-17-92 expression is highly elevated in malignant cells [27, 53–55]. Results showed that miR-17-92 targets phosphatase and tensin homolog (PTEN, tumor suppressor) and Bim (proapoptotic molecule) mRNA directly resulting in lymphoproliferative and autoimmune diseases [56]. In a current study from our lab on microRNA abnormalities in NZB/NZW F1 lupus model by using type I and type III interferons (IFN-*α* and IFN-*λ*) as exogenous disease accelerators, we identified upregulation of several microRNAs correlated to disease severity, yet not with the IFN treatment. MiR-15a was one of the most significant elevated microRNAs as autoimmunity developed in these mice and the level of splenic miR-15a was correlated to the level of anti-dsDNA IgG, in addition, the cellular level of miR-15a was also reflected in the plasma (manuscript accepted for publication).

## 5. MicroRNAs in Hyperimmunoglobulinemias

Multiple myeloma (MM) is characterized by a clonal expansion of plasma B cells in the bone marrow or in extramedullary sites which results in high levels of monoclonal immunoglobulins in the serum [57]. Cytogenetic abnormalities are present in many MM cases, characterized by either hyperdiploidy with the presence of trisomies of odd chromosomes or nonhyperdiploidy with chromosomal aberrations and translocations involving the IgH locus on chromosome 14. In addition to these advancements in understanding MM pathogenesis, studies on the role of microRNAs in recent years have shown them to be key players in MM development not only in the sustenance of malignant cells but also in the initiation of malignancy due to methylation of microRNAs that function as tumor suppressors. Studies investigating microRNAs in MM began in 2007 with the discovery that interleukin 6 (IL-6) indirectly induces the transcription of miR-21 through signal transducer and activator of transcription 3 (STAT3) transcription in the human myeloma cells line. The same upstream enhancer controls miR-21 and STAT3 transcription, and STAT3 controls the transcription of survivin, Bcl2, and Mcl-1. Thus, Stat3 exerts its antiapoptotic affect through the induction of miR-21 [58]. A microRNA microarray analysis in 20 myeloma samples revealed that miR-335 and miR-342-3p were upregulated and may be involved in plasma cell homing and other interactions in the bone marrow [59]. Subsequent studies uncovered various microRNAs that are key players in MM. For example, miR-106b-25 cluster, miR-181a/b, and miR-32 target a histone acetyltransferase, P300/CBP-associated factor (PCAF) that reversibly acetylates transcriptional regulators including p53, thus accounting for the low levels of PCAF observed in MM cells. Also miR-17–92 downregulates Bim, a proapoptotic molecule, and miR-19a/b target SOCS-1, a silencer of the STAT3, thus enhancing the oncogenicity of MM cells [60, 61]. As seen in CLL, miR-15a/16-1 is seen to be downregulated in multiple myeloma [62]. Normally encoded within the DLEU2, a gene frequently deleted in lymphocytic leukemia, miR-15a/16-1 activity is central to the antiproliferative activity of DLEU2 [63], as miR-15a/16-1 inhibits cyclinD1, cyclinD2, and CDC25a [62]. Also, several microRNAs have been seen to target the p53 gene. For example, miR-25 and miR-30d are increased in MM and target the 3′UTR of the p53 gene [64]. Also, MM cells have low levels of miR-192, miR-194, and miR-215 which targets MDM2, a p53 antagonist, thereby lowering p53 levels and increasing oncogenic potential [65]. Moreover, the promoter of miR-34b/c, a transcriptional target of p53 was found to be hypermethylated and thus inactivated in multiple myeloma cell lines. Such epigenetic modifications are observed to be causal in other microRNAs as well. Hypermethylation of the promoters of various tumor suppressors such as miR-124-1 (a target of CDK6) [66], miR-203 (a target of cyclic-responsive element-binding protein which increases proliferation) [67], and miR-29b (a target of Mcl-1 which antagonizes IL-6) increase the tumorigenicity of myeloma cells [68]. Extranodal marginal zone lymphomas are most associated with mucosal-associated lymphoid tissue (MALT) and are characterized by clonal proliferation of plasma cells that produce the immunoglobulin A isotype. Investigations involving microRNAs have found the miR-203 promoter to be hypermethylated in samples of gastric lymphoma, and this microRNA targets the c-abl1 oncogene, thus enabling tumor growth and proliferation [69]. In addition, miR-150 and miR-155 were upregulated, while miR-184, miR-205 and miR-200a/b/c (which targets cyclin E2) were downregulated [70]. In another hyperimmunoglobulin disorder, Immunoglobulin A nephropathy (IgAN) is characterized by the deposition of immune complexes in the kidney mesenchyme causing renal injury and usually coincides with mucosal infections [57]. These immune complexes are composed of IgA1 molecules that are galactose-deficient, causing a conformational change in the molecule, and autoantibodies (IgA or IgG) form to its exposed epitopes [71]. Since miR-155 and miR-146 are involved in B lymphocyte development, their levels were examined in 43 IgAN biopsy specimens and urine samples. The results showed that miR-146 and miR-155 were high in IgAN biopsy and urine sediment, suggesting their role in IgAN pathogenesis [72].

## 6. MicroRNAs in Acute Myeloid Leukemia (AML) 

MicroRNAs (miRNAs) have been well studied in various cancers including leukemias [73, 74]. Acute myeloid leukemia (AML) is a hematopoietic progenitor cell-originated malignant disorder affecting the myeloid lineage, which could be classified into subtypes based on the differentiation stages of the malignant cells found in peripheral blood and in bone marrow [75]. Among various symptoms and manifestations identified in association with AML, one of the most common characteristics involved in ~50% of AML patients is a group of cytogenetic abnormalities, which is considered to be contributing to the disease heterogeneity and with prognostic significance [76]. Other AML patients without detectable chromosomal abnormalities may display mutations or dysregulations in specific genes, a signature ubiquitously found in cancers [77–79]. MicroRNA signatures in AML have been sought, and many groups of researchers performed large-scale profiling of miRNA expression in different populations of AML patients. In the first study where AML patient samples were compared to acute lymphoblastic leukemia (ALL), both groups with similar chromosomal alterations, 27 miRNAs were reported to be different between the two groups [80]. Importantly, miR-146a was inversely correlated to overall survival in both AML and ALL [81]. However, these studies focused on miRNA profile distinguishing AML from ALL, which was not sufficient for understanding the abnormalities of miRNAs expression exclusive to AML. 

Another study compared 122 AML samples to CD34+ cells from 10 normal controls. Among the 122 AML samples, 60 cases were untreated and 54 relapsed or refractory [82]. By microarray profiling, 26 microRNAs were downregulated in AML samples. Several of these downregulated miRNAs in AML were also underexpressed in mature myeloid cells suggesting that miRNAs related to the differentiation patterns in AML (miR-126, miR-130a, miR-93, miR-125a, and miR-146). In correlating cytogenetic abnormalities with miRNA expression, 14 downregulated and 8 upregulated miRNAs were associated with 11q23 translocation versus all other AML, including the downregulation of miR-196 and miR-15a, and overexpression of miR-21 in *t*(6; 11) with worse prognosis [82]. In AML patients (*n* = 36) achieving complete remission the levels of miR-15a/16 were upregulated. Subsequently, in 2 patients in which relapse occurred, miR-15a decreased. All-trans retinoic acid (ATRA) *in vitro* treatment in AML cell lines and primary leukemic cells induced miR-15a/16 upregulation, in addition, miR-15a/16 enhanced the effects of ATRA inducing leukemic cell differentiation[83].Despite the poor overall survival of these AML patients, the study showed several associations between miRNA expression and the outcome of patients, especially the overexpression of miR-199a and miR-191, identified in AML with trisomy 8 and associated with poor outcome. This study was the first to identify the distinct miRNAs profile between AML patients and normal control, and the subsets of miRNAs related to cytogenetic groups and disease outcome [82]. 

Almost at the same time, a study with 215 heterogeneous AML samples was performed to demonstrate the signatures of miRNAs expression in cytogenetic and molecular subtypes [84]. A group of upregulated miRNAs were prominent in *t*(15;17) cases. In contrast, *t*(8;21) was characterized by downregulated miRNA alterations, for example, tumor suppressor let-7. In molecular subgroups of AML, nucleophosmin *(NPM1)* mutations, which represent the most common molecular abnormality in AML, are associated with overexpression of homeobox genes (*HOX*) [85]. Upregulation of miR-10a, miR-10b, miR-196a and miR-196b, was identified in AML carrying *NPM1 *mutations, and these miRNAs were located within the *HOX *genes. Although miR-196a directly targets *HOXB8* mRNA [86], the upregulated miR-196a in this AML subgroups may represent a breakage in the regulation loop between miRNAs and *HOX* genes [84]. Consistent with other studies, miR-155 was significantly upregulated in AMLs with internal tandem duplications of Flt3*(FLT3-*ITD), corroborating the oncogenic effect of miR-155 in myeloid cells in addition to such effects in lymphoid lineages [84, 87, 88]. In comparing AML to normal CD34+ cells, upregulation of miR-21 in AMLs was found, consistent with other studies and further strengthening the importance of miR-21 in AML [82, 84]. 

Interestingly, in an analysis of AML subgroups the *t*(8;21) and inv(16) were grouped together by miRNA profile, supporting the notion that both subgroups belong to core-binding factor (CBF) AMLs, suggesting some common pathways shared by CBF-AMLs [89]. Overexpression of miR-224, miR-368, and miR-382 was restricted to the *t*(15;17) samples, while miR-17-92 cluster was overexpressed exclusively in mixed-lineage leukemia (MLL) rearrangements [89]. In addition, in a study of 100 AMLs, comparing leukemic samples to normal bone marrow, miR-155 and miR-181a were upregulated [90]. MiR-181a has been reported to target p27^Kip1^ in AML cell lines, resulting in an abrogation of 1, 25-dihydroxyvitamin D3 (1,25D) induced differentiation in AML cell lines [91]. A recent study classified AML cases into favorable, moderate, and poor as the predicted outcome according to the karyotype. MiR-181a high expression was suggested to be associated with better-risk groups suggesting a potential therapeutic approach involving manipulation of miR-181a level in AML patients. In contrast to elevated miR-181a as favorable prognostic factor, miR-155 upregulation predicts poor prognosis in AML [92].

## 7. MicroRNAs in Chronic Lymphocytic ****Leukemia (CLL)

CLL is characterized by the accumulation of malignant B-1 cells (CD5^+^CD19^+^CD20^dull^CD23^+^IgM^dull^) in peripheral lymphoid organs, bone marrow, and peripheral blood [93]. It accounts for 30% of all leukemias in the Western world, making it the most common lymphoid malignancy with mainly elderly with disease. CLL is broadly classified into aggressive (Zap70^hi^-unmutated IgH) and indolent (Zap70^low^-mutated IgH) [94]. CLL cells have genomic instability, chromosomal alterations and have several characteristic genetic abnormalities. Prominent among them are 11q23 deletions (ATM; miR-34b/c cluster), trisomy 12 (increased MDM2), 17p deletion (TP53), and 13q14 deletions (miR-15a/16-1) [95]. Dysregulation of several microRNAs like miR-15a/16-1, miR-34 cluster, miR-155, miR-29, and miR-181b has been implicated in the pathogenesis of CLL. The most common genetic abnormality in CLL patients is the deletion of 13q14 region (50–60% of CLL cases) that encodes a crucial microRNA locus, *miR-15a/16-*1 [30, 96]. Decreased miR-15a/16-1 confers a growth advantage as these microRNAs target key cell cycle regulatory and antiapoptotic proteins such as cyclin D1 and Bcl2 [97, 98]. It is interesting to note that NZB mice (spontaneously occurring mouse model of CLL) also exhibit a 50% reduction in the level of miR-15a/16-1, that is associated with a point mutation and deletion in the 3′ flanking region of miR-16-1 [99]. Moreover targeted deletion of the miR-15a/16-1 locus or a larger surrounding minimal deleted region (MDR) led to the development of CLL in mice, further confirming the tumor suppressor function of this locus [32]. Other microRNAs are abnormal in CLL including miR-29 and miR-181, which target Tcl1, a gene that is highly elevated in aggressive CLL [100]. MiR-29 expression is decreased in aggressive CLL, while it is increased in indolent CLL as compared to normal volunteers [100, 101]. Thus, the same microRNA can function as both an oncogene and as a tumor suppressor in CLL. MiR-34a/b/c is decreased in patients with 11q deletions. Normally, upon transactivation by TP53, miR-34 expression would result in decreased Zap70 [102]. MiR-34a has also been shown to target E2F1 and B-Myb oncogenes in CLL as well as AML [103]. MiR-155, miR-150, and miR-21 expression is increased in B-CLL cells as compared to normal B cells [23, 104]. Increased miR-155 levels are associated with increased Zap70 expression and faster progression. v-Myb is found to be elevated in CLL patients, and it stimulates the miR-155 host gene [105]. The oncogenic potential of miR-155 is further supported by the development of B-cell malignancies in E*μ*-mmu-miR-155 transgenic mice [34]. Using a poorly understood mechanism, microRNAs are secreted into body fluids such as serum and urine, and their levels can be used as noninvasive biomarkers for diagnosis and monitoring of cancer and various other diseases [106, 107]. In a recent, study it was shown that elevated miRNA levels in serum may offer early CLL detection and differentiation between Zap70 status [108]. The authors further concluded that increased expression of miR-150, miR-29a, miR-222, and miR-195 can be used as a highly sensitive diagnostic test for CLL.

## 8. Conclusion

**Figure 1:**
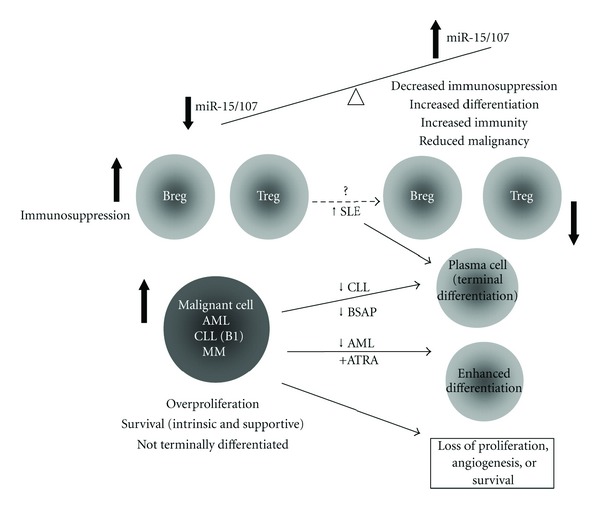
Schematic mechanism of miR-15/107 family alterations in hematopoietic disorders. Decreased expression of miR-15/107 family members is found in malignant cells from AML (acute myeloid leukemia), CLL (chronic lymphocytic leukemia), and MM (multiple myeloma) patients. The underexpression of miR-15/107 may also contribute to increased immunosuppressive regulatory B (Breg) and T cells (Treg), which further promote the expansion and survival of malignant cells. In contrast, with increased miR-15/107, there may be a loss of immunosuppression that leads to SLE (systemic lupus erythematosus) development and antitumor responses. In the therapeutically induced differentiated AML cells, and terminally differentiated B cells, plasma cells (with decreased B-cell-specific activator protein (BSAP), the negative regulator of miR-15a/16-1), miR-15/107 family members would be upregulated, leading to the loss of malignant potential and an increase in differentiation function (SLE).

In this paper, a variety of blood disorders were discussed in terms of microRNA abnormalities observed. One microRNA family of interest stood out as a potential regulator of cell fate (Table 1 and Figure 1). Recently, miR-15 family members (miR-15a/b, miR-16, miR-103, miR-107, miR-195, and miR-497) have been grouped together due to their identical “AGCAGC” sequence at 5′ end “seed region (nucleotides 2–7)” [109, 110], which offers this miRNA group various overlapping functions in gene-regulatory pathways and disease scenarios, especially in cancers. MiR-15/107 gene group could be upregulated by tumor suppressor p53 [102], altered by various cell stress [111–115], or inhibited by Myc [116, 117]. A broad spectrum of mRNAs is targeted by miR-15/107, importantly, miR-15/16 paralogs regulate cell cycle via targeting of Cyclin D1 [97] and induce apoptosis via targeting of Bcl-2 [118], and miR-107 also induces cell cycle arrest [119]. The tight involvement of miR-15/107 in cell growth and cell fate control, and their upstream regulators, such as p53 and Myc, which by themselves are important players in tumorigenesis [120, 121], revealed critical mechanisms for abnormalities in cancer development, including leukemias. Indeed, all members from miR-15/107 group have been identified to be altered in various tumor cells [122–125]. Specifically, underexpression of miR-15a/16 as a result of deletion or mutation of *mir-15a/16* loci has been linked to the pathogenesis of CLL [96, 99, 118], similarly in AML and MM, where the downregulation of miR-15a/16 was associated with the loss of control for malignant cells differentiation and proliferation [62, 83]. In contrast, in SLE which is characterized by elevated plasma cell differentiation contributing to increased autoantibody production [126, 127], splenic miR-15a was increased, and this was significantly correlated with autoantibody levels in lupus-like autoimmune mouse model (manuscript accepted for publication), suggesting a role of miR-15a upregulation in cell cycle arrest in order for plasma cell differentiation. 

Future directions may be directed toward stem cell transplantation for many of these blood disorders. Cellular transplantation therapy holds a huge potential for a variety of degenerative, genetic, and malignant conditions treatment. Hematopoietic stem cell transplantation is the most widely used form of such a therapy, but many patients do not benefit from that because of the lack of a suitable HLA-matched donor [128]. In this sense, patient-specific autologous pluripotent stem cells generation would provide a great opportunity to combine gene therapy with autologous cell transplantation to treat different human conditions including hematological disorders such as AML. For this reason, robust protocols for the generation of safe autologous induced pluripotent stem (iPS) cells are strongly needed. To this end, microRNAs represent an attractive tool for both iPS generation efficiency enhancement and gene targeting approaches. It is known that expression of embryonic stem (ES) cell-specific microRNAs such as miR-294 promotes iPS cells induction from somatic cells [129]. Recently, it has even been demonstrated that the expression of miR-302/367 cluster can directly reprogram mouse and human somatic cells to a pluripotent stem cell state in the absence of the commonly used reprogramming factors [130]. Alternatively, inhibition of tissue-specific miRNAs would also enhance iPS generation, which has been confirmed by antisense silencing of a prodifferentiation let-7 miRNA [131]. Another application of microRNAs lies in promoting patient-specific iPS differentiation towards the required cell lineage, for example HSC expansion. MiR-145 has been shown to induce ES cell differentiation by inhibiting the expression of Sox2, Oct4, Klf4, and c-Myc, key reprogramming factors, and led to an increase of HSC number *in vivo* by more than 8 fold [132–134]. Nevertheless, HSC expansion from iPS cells by means of microRNAs needs to be further developed. Hopefully, the recently achieved success in the production of iPS cells with the use of miRNAs will pave the way for successful *in vitro* expansion of HSCs with miRNAs.

## Figures and Tables

**Table 1:** MiR-15/107 group involvement in common blood disorders^§^.

Blood disorders	MiR-15/107 group alterations	Abnormalities associated	Effects of miRNAs
AML	MiR-15a/16 decreased in *t*(11q23) AML patients [82], elevated in patients with complete remission, and decreased with relapse [83]		MiR-15a/16 enhanced ATRA effects inducing AML cell differentiation [83]

APL	MiR-15a/16 upregulation and miR-107 downregulation in a cohort of APL patients [135]		Patients showed increased miR-15/107 during remission, and miR-15/107 upregulation was induced by ATRA *in vitro* in APL cells [135]

	MiR-15a/16 underexpressed in CLL patients with 13q14 deletion and NZB mice (CLL model) [96, 99]	Uncontrolled B-1 cell proliferation [97]	Overexpression of miR-15a/16 in CLL murine model resulted in exclusive elimination of malignant B-1 cells [136]
CLL	MiR-195 upregulation reported from a study of 9 CLL patients compared to normal controls [137]		Not determined
	MiR-107 downregulated in CLL patients [138]		Underexpression of miR-107 resulted in overexpression of oncogenic PLAG-1 protein [138]

MM	MiR-15a/16 decreased in MM patients [62]	Uncontrolled clonal plasma cell proliferation and proangiogenesis in bone marrow [62]	MiR-15a/16 targeted cell cycle regulators, inhibited NF-*κ*B pathway, and downregulated proangiogenic genes [62]

SLE	miR-15a upregulated in spleen cells from NZB/NZW F1 mice (SLE model), when disease fully developed (manuscript accepted for publication)	Elevated autoreactive antibody producing cells terminally differentiated plasma cells	MiR-15a enhanced plasma cell differentiation

^§^AML: acute myeloid leukemia; APL: acute promyelocytic leukemia; CLL: chronic lymphocytic leukemia; MM: multiple myeloma; SLE: systemic lupus erythematosus; PLAG-1: pleomorphic adenoma gene.

## Abbreviations

miRNA/miR: MicroRNA
AML: Acute myeloid leukemia
ALL: Acute lymphoblastic leukemia
CLL: Chronic lymphocytic leukemia
SLE: Systemic lupus erythematosus
IgAN: Immunoglobulin A nephropathy
MM: Multiple myeloma
TLR: Toll-like receptor
IFN: Interferon
3′UTR: 3′untranslated region
NZB: New Zealand black
ATRA: All-trans retinoic acid
HOX: Homeobox genes
NPM: Nucleophosmin
FLT3-ITD: Internal tandem duplications of Flt3
CBF: Core-binding factor
iPS cell: Induced pluripotent stem cell
HSC: Hematopoietic stem cells
ES cell: Embryonic stem cells.
